# Diagnostic Concordance Across Text and Image Workflows in Nine Consumer Multimodal Artificial Intelligence Systems for Cutaneous Vascular Anomalies: A Cross-Sectional Audit

**DOI:** 10.3390/diagnostics16193100

**Published:** 2026-09-24

**Authors:** Guoyong Wang, Hui Bi, Ye Zhang, Weixin Wang, Yingjie Zhu, Chaonan Wang, Xiaonan Yang

**Affiliations:** 1 Department of Hemangioma and Vascular Malformation, Plastic Surgery Hospital, Chinese Academy of Medical Sciences and Peking Union Medical College, 33 Badachu Road, Shijingshan District, Beijing 100144, China; 2 Department of Internal Medicine, Plastic Surgery Hospital, Chinese Academy of Medical Sciences and Peking Union Medical College, 33 Badachu Road, Shijingshan District, Beijing 100144, China

**Keywords:** artificial intelligence, cutaneous vascular anomalies, diagnostic concordance, multimodal systems, dermatology, clinical decision support

## Abstract

**Background/Objectives**: The reliability of widely accessible multimodal artificial intelligence systems for rare or complex cutaneous vascular anomalies is uncertain. We evaluated diagnostic concordance and expert-rated management outputs across clinical-summary text, clinical-summary text plus images, and image-only workflows. **Methods**: In this cross-sectional audit, a purposively assembled, nonconsecutive challenge set of 511 published cases was evaluated with nine consumer web systems in new-conversation first-response sessions (13,797 final score records). Outputs were classified as top-1 concordance with the source-reported diagnosis, differential-list-only inclusion, or omission. Two vascular-anomaly specialists independently rated expected management appropriateness and safety on 5-point scales. Paired comparisons used exact McNemar, Friedman, and paired Wilcoxon tests. **Results**: Image-only top-1 concordance ranged from 0.4% to 7.0%, with omission rates of 71.0% to 97.8%; corresponding clinical-summary-text ranges were 32.9% to 53.0% and 11.5% to 29.4%. Image-only performance was lower than both text-containing workflows for every system. Text-plus-image workflows were not consistently superior to text alone. **Conclusions**: These date-stamped workflow contrasts do not estimate real-world diagnostic accuracy or isolate the causal contribution of images. They support preservation of clinical context and specialist oversight.

## 1. Introduction

Infantile hemangiomas range from self-limited lesions to those requiring early risk-stratified treatment [1,2]. Cutaneous vascular anomalies comprise tumors and malformations with heterogeneous biology, appearance, natural history, and complications [3,4].

Diagnosis and management may require clinical examination, imaging, pathology, or molecular findings. Severe infantile hemangiomas and lymphatic and venous malformations follow distinct care pathways [5,6,7]. Sclerotherapy and emerging PI3K/AKT/mTOR-targeted therapy illustrate disease-specific treatment [8,9]. Rare tumors such as kaposiform hemangioendothelioma may cause life-threatening coagulopathy [10,11]. Sturge–Weber syndrome may couple cutaneous findings with neurologic disease [12]. Complex cases may benefit from multidisciplinary care and rare-disease networks [5,6,7,13].

Multimodal artificial intelligence models can integrate text and images and are being explored for medical applications [14,15]. Large language models have demonstrated broad clinical knowledge and diagnostic capabilities [16,17]. Generalist biomedical and pathology systems further show cross-modal integration [18,19]. However, benchmark performance does not establish reliability in a rare dermatologic domain. Dermatology-specific systems such as SkinGPT-4 and PanDerm demonstrate the potential value of domain-oriented training [20,21].

Consumer systems may also retrieve or recall information from public sources rather than rely only on the submitted input. Retrieval-augmented generation can ground responses in external knowledge, but retrieval noise, domain shift, incomplete provenance, and limited transparency can compromise healthcare reliability [22]. These concerns are especially relevant when evaluation cases originate from published reports that may have been present in training corpora or accessible to platform retrieval.

Systematic reviews show that artificial intelligence performance in dermatology varies by disease class, image type, comparator, and external-validation setting [23,24]. Foundation-model translation introduces additional technical and safety concerns [25]. CLEAR Derm emphasizes transparent reporting of image-based evaluations [26]. Population diversity can affect model performance [27], and patient-level clinical context should be considered in evaluation [28]. A recent JAAD International comparison of GPT-4 with vision and dermatologists supports direct assessment of consumer visual systems [29]. Systematic evidence in rare or complex cutaneous vascular anomalies remains limited. We therefore conducted a date-stamped cross-sectional audit of nine systems across clinical-summary-text, text-plus-image, and image-only workflows. Because information content and prompt instructions differed, comparisons were interpreted as workflow-level contrasts rather than causal estimates of image effects.

## 2. Materials and Methods

### 2.1. Study Design and Reporting

This cross-sectional, simulation-based audit used published case reports and consumer web interfaces. Reporting followed STARD-AI where applicable and CLEAR Derm for image-based evaluation [26,30]. Broader reporting considerations drew on TRIPOD + AI [31].

The unit of analysis was the case–system–workflow interaction. The final score-level dataset contained all 511 × 9 × 3 records, with no missing score outcomes or duplicate case–system–workflow records.

### 2.2. Case Identification and Source Material

PubMed was searched for English-language case reports concerning cutaneous vascular tumors, vascular malformations, and related syndromes. The search had no date restriction and was completed before model testing; the exact final search date was not retained. Reports were eligible when they described a diagnostically challenging or clinically meaningful presentation and contained sufficient history, examination, and ancillary-test information. Guoyong Wang screened the retrieved literature and selected the cases. Reports outside the target clinical scope, reports for which full text could not be obtained, and reports with critical information missing, animal cases, or duplicate content were excluded. The search identified 1223 records, and the final challenge set comprised 511 cases. The number of included source reports and reason-specific exclusion counts could not be verified from the available records. The assembled set also included vascular-appearing mimics and related nonvascular conditions, as described in the post hoc case-spectrum summary. The cases formed a purposive, nonconsecutive challenge set for stress testing. The final diagnosis reported in each source article served as the comparator and was not independently re-established. The study flow is shown in Figure 1.

Clinical-summary-text inputs were assembled from discrete source fields for chief complaint, present illness, past medical history, physical examination, and ancillary examinations. Bibliographic titles, authors, journal names, figure captions or legends, explicit diagnosis fields, dedicated pathology and molecular-result fields, treatment, treatment response, and follow-up were not intentionally supplied. However, no case-level de-cueing audit was retained; diagnostic clues could remain within narrative fields or inside source images or annotations. Images were obtained from figures in the same reports and included clinical photographs and radiologic images. For each case, the same local image set was used across systems whenever accepted. Original format and resolution were preserved when possible, and no systematic cropping, annotation, contrast adjustment, denoising, or lesion marking was performed. Platform-side image processing was not observable.

To describe case spectrum, source-report diagnostic labels were grouped post hoc using a pragmatic label-term rule into four broad categories. This descriptive grouping was not an independent clinical or ISSVA reclassification and was not used for subgroup-performance analysis.

### 2.3. Systems, Workflows, and Prompting

Nine consumer-facing systems were accessed through web interfaces during January and February 2025: ChatGPT 4o, Claude 3.5 Sonnet, Gemini 2.0, Copilot, Wenxiaoyan, Kimi, Doubao, Tongyi, and Baixiaoying. Research application programming interfaces were not used. The archived records do not consistently preserve whether each 2025 session used a free/basic or paid subscription tier, so account tier cannot be compared. Exact per-interaction timestamps and a valid chronological variable were not uniformly preserved. Consequently, progressive improvement or ‘autolearning’ was not assessed. Platform labels describe the tested interfaces; immutable backend versions, routing, system prompts, temperature, top-*p*, and seed were unavailable.

Each case was evaluated under three workflows: clinical-summary text (H, the original dataset code), clinical-summary text plus images (H + I), and images only (I). Prompts were fixed across systems within each workflow but were modality-tailored rather than identical across workflows: prompts containing images requested a lesion description before diagnosis, whereas the text-only prompt did not. The exact prompts are reproduced in the Supplementary Methods. Each interaction used a new conversation and one first response; no regeneration, follow-up questioning, or system-specific prompt optimization was used. New conversations and available privacy/training opt-out settings reduced visible within-thread carryover and were intended to prevent training reuse, but they do not exclude cross-chat memory, personalization, account or browser state, platform retrieval, or temporal backend drift.

### 2.4. Outcomes and Rating

Ye Zhang and Weixin Wang, both physicians with more than 10 years of clinical experience, categorized diagnostic outputs against the source-reported final diagnosis. Each output was assigned one mutually exclusive category: top-1 concordance (the source-reported diagnosis was ranked first), differential-list-only inclusion (it appeared only in the differential diagnosis list), or omission (it was not mentioned). The clinicians used clinical judgment to assess whether synonymous or alternative vascular-anomaly terminology referred to the source-reported diagnosis. Ambiguous rankings and compound diagnostic wording were assessed through the same clinical judgment process. When Ye Zhang and Weixin Wang were uncertain about a diagnostic category or disagreed on its assignment, Xiaonan Yang made the final decision. The final category codes are provided in Supplementary Data S1.

Two vascular-anomaly specialists, Ye Zhang and Weixin Wang, independently rated each management recommendation using prespecified 5-point scales. The original dataset called the first dimension ‘effectiveness’; because no patient outcomes were observed, it is reported here as expected management appropriateness. Higher scores indicated closer alignment with accepted practice and fewer safety concerns. The arithmetic mean of the two ratings was used for workflow comparisons. Masking to system and workflow labels was incompletely documented.

### 2.5. Statistical Analysis

Proportions were calculated for each diagnostic category by system and workflow. Within each system and diagnostic endpoint, two-sided exact McNemar tests compared H + I with H, I with H, and I with H + I. Paired odds ratios were calculated from discordant pairs; 0.5 was added to both discordant counts when either count was zero for odds-ratio and interval estimation. Confidence intervals are pointwise, unadjusted 95% Wald intervals on the log-odds-ratio scale. The exact McNemar *p*-values were calculated from the uncorrected discordant counts and Bonferroni-adjusted across the three contrasts within each system-endpoint family. Statistical significance was assessed using the adjusted *p*-values.

Management ratings were compared across workflows within each system using Friedman tests. When the omnibus result was significant, paired Wilcoxon signed-rank tests without continuity correction were Holm-adjusted within each combination of system and management dimension (three workflow contrasts per family). Kendall W, standardized effect size r (the absolute standardized Wilcoxon statistic divided by the square root of the number of nonzero paired differences), paired mean differences, and percentile bootstrap 95% confidence intervals from 2000 case-level resamples were calculated. Interrater reliability was summarized using Cronbach’s alpha and a two-way mixed-effects, consistency, single-rater intraclass correlation coefficient [ICC(3,1)]. Reliability was summarized overall, by workflow pooling the nine systems, and separately by system pooling the three workflows. No formal sample-size calculation was performed; all 511 included cases were evaluated. Original analyses were performed in R version 4.3.1, and score-derived numerical results were independently checked from the final score-level dataset during revision.

### 2.6. Ethics and Data Governance

The study used curated case summaries and images derived solely from publicly published reports and involved no patient recruitment, intervention, contact, or access to nonpublic medical records. Public-source images were uploaded as evaluation inputs under available local/privacy controls, with training reuse disabled where the platform offered that option. Commercial-platform retention, secondary processing, cross-session memory, and deletion were not independently verified. Institutional review board approval was not obtained. Source articles and images were treated as third-party materials and are not redistributed; no source patient image or model-response text is reproduced in the manuscript, Supplementary Materials, or public release. The public score dataset uses numeric case identifiers.

## 3. Results

### 3.1. Study Sample and Diagnostic Concordance

The 511 cases were tested across nine systems and three workflows, yielding 13,797 final case–system–workflow score records. The post hoc label-based spectrum comprised 241 (47.2%) vascular/endothelial tumors or reactive proliferations, 137 (26.8%) syndromic or complex vascular/overgrowth presentations, 56 (11.0%) vascular malformations or related vessel anomalies, and 77 (15.1%) vascular-appearing mimics or related nonvascular conditions. These post hoc categories summarize the diagnostic spectrum of the challenge set. Diagnostic results are summarized in Table 1. Under the image-only workflow, top-1 concordance ranged from 0.4% to 7.0%, differential-list-only inclusion from 1.8% to 21.9%, and omission of the source-reported diagnosis from 71.0% to 97.8%. Under clinical-summary text, the corresponding ranges were 32.9% to 53.0%, 32.7% to 47.2%, and 11.5% to 29.4%.

For every system, image-only input had lower top-1 concordance than clinical-summary text (paired odds ratio range, 0.0019–0.069; all adjusted *p* < 0.001) and higher omission (paired odds ratio range, 22.07–166.00; all adjusted *p* < 0.001). The same direction was observed for images only versus clinical-summary text plus images (top-1 odds ratio range, 0.0020–0.062; omission odds ratio range, 20.08–125.67; all adjusted *p* < 0.001).

Exploratory H + I versus H comparisons were heterogeneous because prompt instructions differed. No statistically significant top-1 difference was detected for ChatGPT 4o or Claude 3.5 Sonnet; top-1 concordance was lower under H + I for the other seven systems. Omission was higher under H + I for eight systems, with no statistically significant difference detected for ChatGPT 4o. Figure 2 displays the paired comparisons.

### 3.2. Management Ratings and Interrater Reliability

Across all 13,797 case–system–workflow interactions, interrater reliability was high for expected management appropriateness (Cronbach’s alpha = 0.947; ICC(3,1) = 0.898) and safety (alpha = 0.915; ICC(3,1) = 0.843). Table 2 presents workflow-stratified estimates pooled across the nine systems (4599 interactions per workflow); ICC(3,1) ranged from 0.727 to 0.912 for appropriateness and from 0.746 to 0.848 for safety. In a separate system-stratified analysis pooled across the three workflows (1533 interactions per system), ICC(3,1) ranged from 0.768 to 0.935 for appropriateness and from 0.807 to 0.862 for safety.

Friedman tests identified workflow differences in appropriateness and safety for every system (all *p* < 0.001; Table 3). Clinical-summary text scores exceeded image-only scores for appropriateness by 0.924 to 2.169 points and for safety by 0.494 to 0.841 points (all Holm-adjusted *p* < 0.001). Differences between H and H + I varied by system. For ChatGPT 4o, mean appropriateness/safety ratings were 4.03/4.14 under H, 4.03/4.14 under H + I, and 3.11/3.30 under I; no statistically significant difference was detected between H and H + I.

## 4. Discussion

This audit found a large and consistent performance gap between image-only workflows and workflows containing published clinical-summary text. Across all nine systems, image-only first responses frequently omitted the diagnosis reported in the source case and received lower offline expert ratings for expected management appropriateness and safety. The direction and magnitude of this finding were more consistent than differences among individual platforms.

The text-containing workflows included condensed clinical history, examination, and ancillary-test information, while images varied in modality and quality. Image-containing prompts also requested a lesion description. Accordingly, H versus H + I estimates a workflow-level contrast; the design does not separate the contribution of images from information content and prompting. The evaluated H + I workflow showed no consistent incremental benefit over H.

The image-only finding has clinical relevance. Vascular tumors and malformations may share surface color or morphology while differing in flow, deep extent, and biological behavior [3,4]. Rare tumors and syndromic disease may carry systemic consequences not evident from one image [10,11,12]. A plausible response can therefore omit a consequential diagnosis. Disease-specific pathways require clinical history and imaging [5,6,7]. Specialist review is multidisciplinary [13]. Domain-specific models may encode visual features differently [20,21], but image-set variation, metadata, and population diversity limit extrapolation to consumer platforms [27,28]. A JAAD International comparison similarly supports task-specific benchmarking [29].

Because the cases originated from public reports, memorization and retrieval may contribute to diagnostic concordance. The consumer interfaces did not expose whether a response relied on internal parametric memory, web search, a retrieval layer, or solely on the submitted prompt. Retrieval-augmented generation can improve grounding when authoritative evidence is retrieved, yet healthcare reviews also identify retrieval noise, domain shift, incomplete provenance, and limited pipeline transparency as reliability risks [22]. Concordance may therefore reflect a combination of clinical reasoning, prior exposure, and retrieval. Future audits should use prospectively assembled or otherwise nonpublic cases, predeclare and control retrieval states, retain citations and retrieval logs where available, and evaluate retriever and generator errors separately.

Management results followed the same workflow pattern. Ratings reflected expert-perceived appropriateness and safety, not patient outcomes. Missing clinical details may obscure disease-specific pathways [5,6,7], and selection of sclerotherapy or targeted therapy depends on subtype and biology [8,9]. Multidisciplinary input may be required [13]. High interrater reliability indicates consistency between the two specialists’ management ratings.

Generalizability is limited by the purposive, nonconsecutive selection of published challenge cases and associated selection and spectrum bias; this design does not estimate real-world diagnostic accuracy. Reason-specific exclusion counts were unavailable. Source diagnoses were not independently re-established, and the post hoc spectrum grouping was label-based. Inputs differed in clinical information, prompts, image modality and quality, and potential diagnostic cues. The study did not include a clinician comparator or prospective patient outcomes.

Run order, timestamps, and masking were incompletely documented. Immutable backend versions, routing, sampling settings, and platform-side image processing were unavailable, and single-response evaluation did not assess repeatability. Complete response text and contemporaneous adjudication records are not supplied, so output-to-score assignments cannot be independently re-adjudicated. Platform retention, secondary processing, and deletion of uploaded public-source images could not be independently verified.

Future studies should combine prospectively assembled cases with information-matched prompts, version-controlled systems, controlled retrieval, randomized run order, repeated generations, clinician comparators, and prespecified governance. Preserving prompts, outputs, adjudication records, and image metadata would improve auditability. Reporting can draw on CLEAR Derm, STARD-AI, and relevant TRIPOD + AI principles [26,30,31].

## 5. Conclusions

In conclusion, image-only workflows in this date-stamped audit showed substantially lower concordance with source-reported diagnoses and lower expert-rated management quality than workflows containing information-rich clinical-summary text. The findings support preservation of clinical context and specialist oversight, while highlighting the need for prospectively assembled, version-controlled, information-matched, and fully auditable studies.

## Figures and Tables

**Figure 1:**
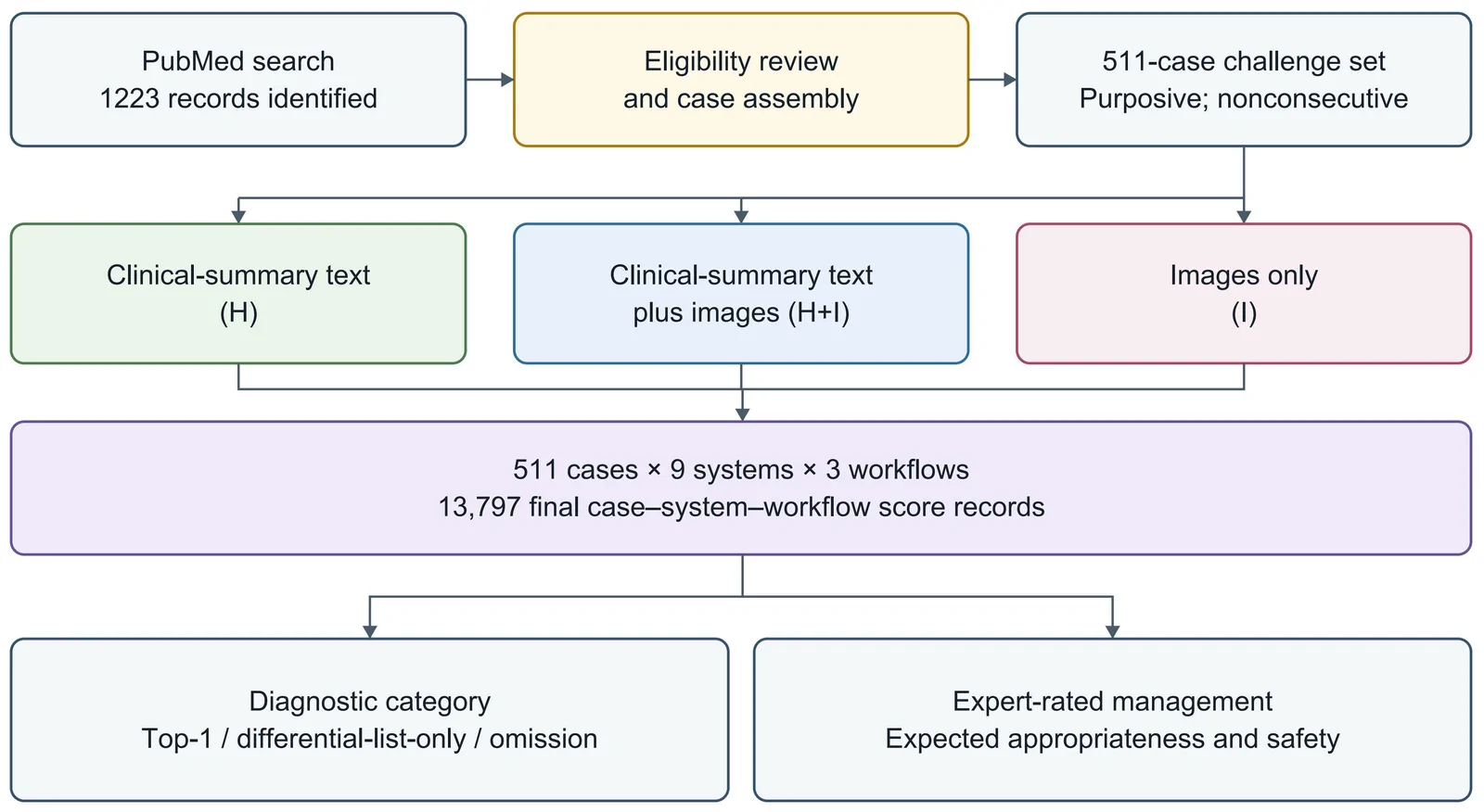
Search, case assembly, and evaluation workflow. Search records (*n* = 1223) and analyzed cases (*n* = 511) are distinct counting units. The cases formed a purposively assembled, nonconsecutive challenge set. H, H + I, and I denote clinical-summary text, clinical-summary text plus images, and images only, respectively.

**Figure 2:**
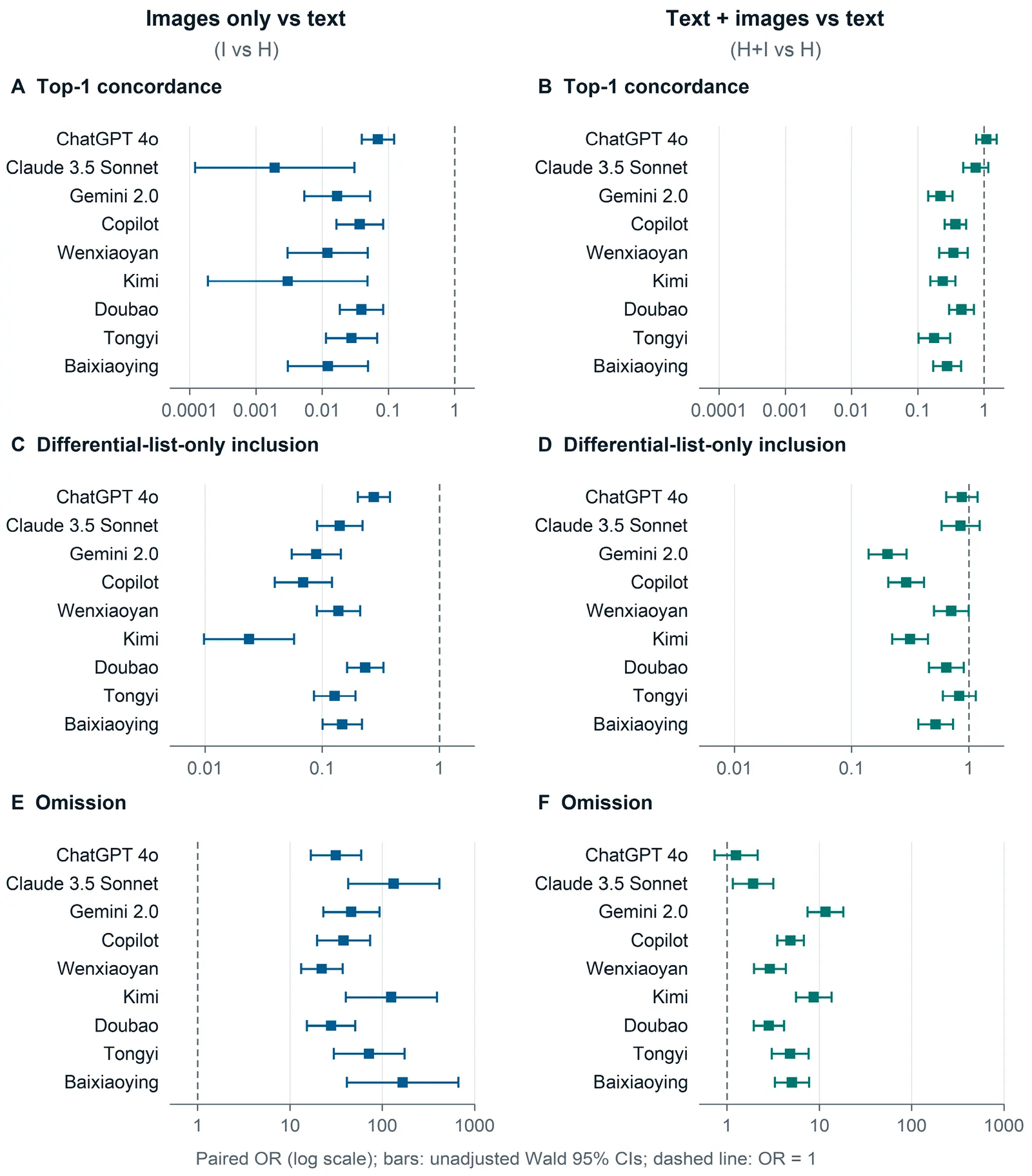
Paired diagnostic-category comparisons from the final score dataset. Forest plots show paired odds ratios and pointwise, unadjusted 95% Wald confidence intervals for I versus H (**left**) and H + I versus H (**right**) across nine systems. For each category, an odds ratio below 1 indicates that the category was less frequent in the first-named workflow; an odds ratio above 1 indicates that it was more frequent. Differential-list-only inclusion is interpreted alongside top-1 concordance and omission. Statistical significance is based on exact McNemar tests with within-family Bonferroni adjustment. The vertical dashed line denotes an odds ratio of 1. H, clinical-summary text; H + I, clinical-summary text plus images; I, images only. (**A**) Top-1 concordance, I versus H; (**B**) top-1 concordance, H + I versus H; (**C**) differential-list-only inclusion, I versus H; (**D**) differential-list-only inclusion, H + I versus H; (**E**) omission, I versus H; and (**F**) omission, H + I versus H. Panels (**A**), (**C**), and (**E**) (blue) show I versus H for top-1 concordance, differential-list-only inclusion, and omission, respectively; panels (**B**), (**D**), and (**F**) (teal) show the corresponding H + I versus H comparisons. Squares denote odds ratios, and horizontal bars denote confidence intervals. Blue denotes I versus H, and teal denotes H + I versus H. Squares show paired odds ratios, and horizontal bars show pointwise, unadjusted 95% Wald confidence intervals.

**Table 1:** Diagnostic concordance across input workflows (N = 511 cases per system–workflow).

System	Clinical-Summary Text (H)	Clinical-Summary Text + Images (H + I)	Images Only (I)
ChatGPT 4o	41.3/47.2/11.5	42.3/45.0/12.7	7.0/21.9/71.0
Claude 3.5 Sonnet	53.0/32.7/14.3	50.7/30.9/18.4	2.0/6.5/91.6
Gemini 2.0	35.8/42.3/21.9	17.6/16.8/65.6	1.4/6.1/92.6
Copilot	32.9/39.1/28.0	20.2/20.2/59.7	2.2/4.7/93.2
Wenxiaoyan	33.1/37.6/29.4	25.2/33.1/41.7	0.8/8.0/91.2
Kimi	32.9/42.1/25.0	17.2/24.7/58.1	0.4/1.8/97.8
Doubao	38.7/36.8/24.5	31.7/30.9/37.4	4.9/12.9/82.2
Tongyi	37.0/43.1/20.0	23.3/40.3/36.4	2.5/8.2/89.2
Baixiaoying	33.7/43.8/22.5	22.9/34.8/42.3	1.8/11.2/87.1

Note: Values are percentages in the order top-1 concordance/differential-list-only inclusion/omission of the source-reported diagnosis. H, H + I, and I are original dataset codes. Category percentages may sum to 99.9% or 100.1% because of rounding.

**Table 2:** Interrater reliability of expected management appropriateness and safety ratings.

Category	*N*	Appropriateness Alpha	Appropriateness ICC(3,1)	Safety Alpha	Safety ICC(3,1)
Overall	13,797	0.947	0.898	0.915	0.843
Clinical-summary text (H)	4599	0.842	0.727	0.899	0.816
Clinical-summary text + images (H + I)	4599	0.952	0.909	0.918	0.848
Images only (I)	4599	0.954	0.912	0.855	0.746

Note: Alpha indicates Cronbach’s alpha. ICC(3,1) is a two-way mixed-effects, consistency, single-rater coefficient. *N* denotes the number of case–system–workflow interactions. Overall estimates pool all nine systems and three workflows (511 × 9 × 3 = 13,797 interactions); each workflow-specific estimate pools all nine systems (511 × 9 = 4599 interactions). Each interaction was rated independently by two specialists. The separate system-stratified analysis pools all three workflows within each system (511 × 3 = 1533 interactions).

**Table 3:** Expected management appropriateness and safety ratings by system and input workflow.

System	H: Appropriateness/Safety	H + I: Appropriateness/Safety	I: Appropriateness/Safety	Appropriateness *p*; W	Safety *p*; W
ChatGPT 4o	4.03 ± 0.76/4.14 ± 0.78	4.03 ± 0.77/4.14 ± 0.78	3.11 ± 0.70/3.30 ± 0.55	*p* < 0.001; 0.416	*p* < 0.001; 0.382
Claude 3.5 Sonnet	4.17 ± 0.83/4.28 ± 0.79	4.05 ± 0.90/4.21 ± 0.81	2.62 ± 0.65/3.53 ± 0.51	*p* < 0.001; 0.678	*p* < 0.001; 0.249
Gemini 2.0	3.85 ± 0.81/3.96 ± 0.81	2.37 ± 1.52/3.44 ± 0.74	2.02 ± 1.08/3.12 ± 0.35	*p* < 0.001; 0.499	*p* < 0.001; 0.364
Copilot	3.79 ± 0.82/3.90 ± 0.82	2.52 ± 1.50/3.51 ± 0.75	1.91 ± 1.04/3.21 ± 0.43	*p* < 0.001; 0.498	*p* < 0.001; 0.227
Wenxiaoyan	3.78 ± 0.81/3.89 ± 0.80	3.29 ± 1.17/3.68 ± 0.79	2.72 ± 0.74/3.21 ± 0.39	*p* < 0.001; 0.381	*p* < 0.001; 0.217
Kimi	3.81 ± 0.80/3.90 ± 0.80	2.57 ± 1.42/3.54 ± 0.73	1.64 ± 0.73/3.41 ± 0.49	*p* < 0.001; 0.628	*p* < 0.001; 0.109
Doubao	3.89 ± 0.83/4.01 ± 0.82	3.45 ± 1.23/3.80 ± 0.84	2.78 ± 0.88/3.26 ± 0.51	*p* < 0.001; 0.388	*p* < 0.001; 0.266
Tongyi	3.88 ± 0.82/3.98 ± 0.80	3.41 ± 1.06/3.70 ± 0.76	2.68 ± 0.85/3.20 ± 0.43	*p* < 0.001; 0.446	*p* < 0.001; 0.305
Baixiaoying	3.83 ± 0.80/3.92 ± 0.80	3.21 ± 1.21/3.62 ± 0.75	2.59 ± 0.84/3.26 ± 0.43	*p* < 0.001; 0.473	*p* < 0.001; 0.208

Note: Values are mean ± standard deviation of the arithmetic mean of two independent ratings. The original data field labeled ‘effectiveness’ is reported as expected management appropriateness. *p* and Kendall W are from within-system Friedman tests across H, H + I, and I.

## Data Availability

The final de-identified diagnostic and management scores, scoring rubrics, and statistical materials are openly available in the Ydata-Vasc511 version 1.1.0 release at https://github.com/wang177777/Ydata-Vasc511/releases/tag/v1.1.0 (accessed on 10 August 2026). The score-only materials support statistical recalculation but do not permit independent re-adjudication of the original output-to-score assignments. Source patient images, full-text articles, platform-history records, complete model-response text, and contemporaneous adjudication records are not included in the manuscript, revision materials, or public release.

## Supplementary Materials

The following supporting information can be downloaded at: https://www.mdpi.com/article/10.3390/diagnostics16193100/s1. Supplementary Methods (eMethods 1–8), including Table S1, “Broad post hoc distribution of source-report diagnostic labels in the 511-case challenge set”; Supplementary Data S1 (final diagnostic and management scoring results).

## Abbreviations

AI, artificial intelligence; CI, confidence interval; CLEAR Derm, Checklist for Evaluation of Image-Based Artificial Intelligence Reports in Dermatology; H, clinical-summary text (original dataset code); H + I, clinical-summary text plus images (original dataset code); I, images only (original dataset code); ICC, intraclass correlation coefficient; OR, odds ratio; STARD-AI, Standards for Reporting Diagnostic Accuracy Studies Using Artificial Intelligence; TRIPOD + AI, Transparent Reporting of a Multivariable Prediction Model for Individual Prognosis or Diagnosis plus artificial intelligence.
