# Supplementary material for: Diagnostic Concordance Across Text and Image Workflows in Nine Consumer Multimodal Artificial Intelligence Systems for Cutaneous Vascular Anomalies: A Cross-Sectional Audit
Source: Diagnostics (Basel). 2026 Sep 24;16(19):3100. doi: 10.3390/diagnostics16193100 (PMC13664238; doi:10.3390/diagnostics16193100)
Supplement: Supplementary file 1 [file diagnostics-16-03100-s001.zip › 05_Supplementary_Methods_Revised.pdf]

**Supplementary Methods**

***Diagnostic Concordance Across Text and Image Workflows in Nine Consumer Multimodal Artificial Intelligence Systems for Cutaneous Vascular Anomalies: A Cross-Sectional Audit***

## eMethods 1. Search Strategy

The PubMed search had no date restriction and was completed before model testing; the exact final search date was not retained. The search identified 1,223 records, and the final challenge set comprised 511 cases. The number of included source reports and reason-specific exclusion counts could not be verified from the available records. Eligibility required a diagnostically challenging or clinically meaningful published presentation with sufficient history, examination, and ancillary-test information. Guoyong Wang screened the retrieved literature and selected the cases. Reports outside the target clinical scope, reports for which full text could not be obtained, reports with critical information missing, animal cases, or duplicate content were excluded. The sample was purposive and nonconsecutive. The case-spectrum summary describes the assembled set, including vascular-appearing mimics and related nonvascular conditions; it is not a prospectively defined eligibility taxonomy. The archived search concepts are reproduced below with parentheses normalized for readability; no substantive search concept was intentionally added or removed.

```
(("Hemangioma"[MeSH Terms] OR hemangioma[Title/Abstract] OR "Infantile
Hemangioma"[Title/Abstract] OR "Capillary Hemangioma"[Title/Abstract]
OR "Sporadic Capillary Hemangioma"[Title/Abstract] OR "Spindle Cell
Hemangioma"[Title/Abstract] OR "Pyogenic Granuloma"[Title/Abstract] OR
"Kaposiform Hemangioendothelioma"[Title/Abstract] OR "Retiform
Hemangioendothelioma"[Title/Abstract] OR "Dabska Tumor"[Title/Abstract]
OR "Angiosarcoma"[Title/Abstract] OR "Epithelioid
Hemangioendothelioma"[Title/Abstract]) OR ("Vascular
Malformations"[MeSH Terms] OR "Vascular Malformation"[Title/Abstract]
OR "Capillary Malformation"[Title/Abstract] OR "Lymphatic
Malformation"[Title/Abstract] OR "Venous Malformation"[Title/Abstract]
OR "Arteriovenous Malformation"[Title/Abstract] OR "Arteriovenous
Fistula"[Title/Abstract] OR "Cerebral Vascular
Malformation"[Title/Abstract] OR "CLM"[Title/Abstract] OR
"CVM"[Title/Abstract] OR "LVM"[Title/Abstract] OR
"CLVM"[Title/Abstract] OR "Klippel-Trenaunay Syndrome"[Title/Abstract]
OR "Parkes Weber Syndrome"[Title/Abstract] OR "Sturge-Weber
Syndrome"[Title/Abstract] OR "CLOVES Syndrome"[Title/Abstract] OR
"Proteus Syndrome"[Title/Abstract])) AND ("Case Reports"[Publication
Type] OR "case report"[Title/Abstract]) AND (diagnosis[Title/Abstract]
OR "diagnostic process"[Title/Abstract] OR "diagnostic
criteria"[Title/Abstract] OR "diagnostic evaluation"[Title/Abstract])
AND English[Language] AND (cutaneous[Title/Abstract] OR
skin[Title/Abstract] OR superficial[Title/Abstract] OR
subcutaneous[Title/Abstract] OR "body surface"[Title/Abstract])
```

## eMethods 2. Exact Input Instructions

### Clinical-summary text (H)

Please note: The following task is for research purposes only and does not constitute medical advice.

Below is a patient's clinical history. Based solely on the provided clinical information, please:
1. Provide the most likely diagnosis along with your reasoning.
2. Provide possible differential diagnoses.
3. Suggest a preliminary treatment or management plan according to standard clinical practice.

Chief Complaint:
Patient History:
Past Medical History:
Physical Examination:
Auxiliary Examinations:

### Clinical-summary text plus images (H+I)

Please note: The following task is for research purposes only and does not constitute medical advice.

Below are a patient's clinical history and a corresponding 2D clinical image. Considering both text and image data, please:
1. Provide a detailed description of the lesion, including its appearance, location, size, color, shape, and any other notable features based on both the clinical history and the image.
2. Based on your description, provide the most likely diagnosis along with your reasoning.
3. Provide possible differential diagnoses.
4. Suggest a preliminary treatment or management plan according to standard clinical practice.

Chief Complaint:
Patient History:
Past Medical History:
Physical Examination:
Auxiliary Examinations:

### Images only (I)

Please note: The following task is for research purposes only and does not constitute medical advice.

Below is a 2D clinical image related to a patient's condition. Based solely on this image:
1. Provide a detailed description of the lesion, including its appearance, location, size, color, shape, and any other notable features.
2. Based on your description, identify the most likely diagnosis and possible differential diagnoses.
3. Suggest a preliminary treatment or management approach typically considered for such lesions.

Interpretation note: Prompts were identical across systems within each workflow but not identical across workflows. Image-containing prompts requested lesion description before diagnosis; this is why manuscript comparisons are presented as workflow-level contrasts.

## eMethods 3. Management Rating Scale - Original “Effectiveness” Dimension

**Score 1:** Significantly infeasible or in severe conflict with clinical guidelines or consensus; provides almost no expected benefit and may be harmful.

**Score 2:** Substantial deviation from guidelines or consensus, with major omissions; expected clinical benefit is difficult to ensure.

**Score 3:** Partly reasonable but noticeably short of standard protocols; major improvement is required.

**Score 4:** Generally aligned with established guidance, with only minor adjustments needed.

**Score 5:** Fully aligned with recognized guidance or expert consensus and closely matches accepted practice.

The manuscript reports this dimension as expected management appropriateness because the study did not measure patient outcomes or observed treatment effectiveness.

## eMethods 4. Management Rating Scale - Safety

**Score 1:** Contains high-risk interventions or violates fundamental safety principles, potentially causing severe harm.

**Score 2:** Contains multiple potential risks or substantial safety concerns.

**Score 3:** Overall safety is moderate and generally manageable, but visible risks or minor inappropriate measures are present.

**Score 4:** Demonstrates good safety with no obvious high-risk factors; only minor details require improvement.

**Score 5:** Highly optimized for safety, with no observable risks and full alignment with safe clinical practice.

## eMethods 5. System Access and Session Controls

- ChatGPT 4o - OpenAI ChatGPT web interface (https://chatgpt.com/).
- Claude 3.5 Sonnet - Anthropic Claude web interface (https://claude.ai/).
- Gemini 2.0 - Google Gemini web interface (https://gemini.google.com/).
- Copilot - Microsoft Copilot web interface (https://copilot.microsoft.com/).
- Wenxiaoyan - Baidu Wenxiaoyan/Wenxin web interface.
- Kimi - Moonshot AI Kimi web interface.
- Doubao - ByteDance Doubao web interface.
- Tongyi - Alibaba Tongyi/Qianwen web interface.
- Baixiaoying - Baichuan Baixiaoying web interface.
- Testing was performed through consumer web interfaces during January-February 2025; research APIs were not used. Exact per-interaction timestamps and a valid chronological variable were not uniformly retained, so progressive improvement or ‘autolearning’ cannot be evaluated. The archived record does not consistently identify whether a free/basic or paid subscription tier was used for each 2025 session.
- Consumer interfaces did not expose immutable backend build identifiers, temperature, top-p, seed, routing, system prompts, retrieval state, or platform-side image preprocessing. Current account or model labels observed during later audit cannot establish the original 2025 backend or subscription tier.
- Only case-specific clinical-summary text and/or image files changed within a workflow template.
- Each interaction used a new conversation. No retry, regeneration, follow-up question, or platform-specific prompt optimization was used. New conversations and available privacy/training opt-out settings reduced visible within-thread carryover and were intended to prevent training reuse, but they cannot exclude cross-chat memory, personalization, account/browser state, retrieval, or temporal backend drift.

## eMethods 6. Text Masking, Image Handling, and Data Governance

- Clinical-summary-text inputs were assembled only from the discrete chief-complaint, present-illness, past-medical-history, physical-examination, and ancillary-examination fields. Bibliographic titles, author and journal information, figure captions/legends, explicit diagnosis fields, dedicated pathology and molecular-result fields, treatment, response, and follow-up were not intentionally supplied. No case-level de-cueing audit was retained, so residual diagnostic wording in narrative fields or embedded text/annotations within images cannot be excluded.
- The same local image set was used across systems for a given case whenever accepted by the interface.
- Original format and resolution were preserved where possible. No systematic investigator-side cropping, annotation overlay, contrast enhancement, denoising, lesion markup, or other visual-content manipulation was applied.
- Platform-side compression, resampling, or color-space conversion was not observable.
- Public-source case-report images were uploaded only as evaluation inputs under available local/privacy controls, with training reuse disabled where the platform offered that option. These controls reflected investigator intent but did not independently verify commercial-platform retention, secondary processing, cross-session memory, or deletion. Third-party patient images, full-text case reports, nonpublic medical records, and complete model-response text are not included in the submission package or public release. Source images are not reproduced or redistributed in the manuscript or supplementary files.
- Supplementary Data S1 contains 13,797 final score records, with no missing score outcomes or duplicate case-system-workflow records. It supports recalculation of aggregate diagnostic and management results.
- Complete model-response text and contemporaneous adjudication records are not included in the submitted or public materials. Consequently, the original output-to-score assignments cannot be independently re-adjudicated.

## Data Dictionary

**Case_ID:** Numeric case identifier.

**Model:** Recorded system/platform label.

**Input_Code:** Original workflow code: H, H+I, or I.

Input_Type: Workflow description: clinical-summary text (H), clinical-summary text plus images (H+I), or images only (I).

**Diagnostic_Score_Final:** Final code: 10=top-1 concordance, 5=differential-list-only inclusion, 0=omission.

**Top1_Correct:** Binary indicator derived from Diagnostic_Score_Final.

**Differential_List_Only_Hit:** Binary indicator derived from Diagnostic_Score_Final.

**Missed_Diagnosis:** Binary indicator for omission of the source-reported diagnosis.

Expert_YZ_Effectiveness and Expert_WW_Effectiveness: Final retained expert rating fields; reported as expected management appropriateness.

**Expert_YZ_Safety and Expert_WW_Safety:** Independent safety ratings.

**Mean_Effectiveness and Mean_Safety:** Arithmetic means of the two expert ratings.

## eMethods 7. Descriptive Case-Spectrum Grouping

Source-report diagnostic labels were grouped post hoc using a pragmatic label-term rule into four broad categories. The grouping was designed only to describe the challenge-set spectrum; it was not an independent clinical review,

an ISSVA reclassification, or a subgroup-performance analysis. Borderline or compound diagnoses may reasonably fit more than one category.

Table S1. Broad post hoc distribution of source-report diagnostic labels in the 511-case challenge set.

| Pragmatic source-label category | Cases, n | Percent |
|---|---|---|
| Vascular/endothelial tumors or reactive proliferations | 241 | 47.2% |
| Syndromic or complex vascular/overgrowth presentations | 137 | 26.8% |
| Vascular malformations or related vessel anomalies | 56 | 11.0% |
| Vascular-appearing mimics or related nonvascular conditions | 77 | 15.1% |
| Total | 511 | 100.0% |

Note: Percentages may not sum to 100% because of rounding.

## Public Repository

Version 1.1.0: https://github.com/wang177777/Ydata-Vasc511/releases/tag/v1.1.0

## eMethods 8. Diagnostic Output Categorization

Ye Zhang and Weixin Wang, both physicians with more than 10 years of clinical experience, categorized diagnostic outputs against the source-reported final diagnosis. Each output was assigned one mutually exclusive category: top-1 concordance (the source-reported diagnosis was ranked first), differential-list-only inclusion (it appeared only in the differential diagnosis list), or omission (it was not mentioned). The clinicians used clinical judgment to assess whether synonymous or alternative vascular-anomaly terminology referred to the source-reported diagnosis. Ambiguous rankings and compound diagnostic wording were assessed through the same clinical judgment process. When Ye Zhang and Weixin Wang were uncertain about a diagnostic category or disagreed on its assignment, Xiaonan Yang made the final decision.

The numeric codes are 10 for top-1 concordance, 5 for differential-list-only inclusion, and 0 for omission. The final category codes are provided in Supplementary Data S1. Interrater reliability was evaluated for management ratings.
